# Potassium Hydroxide Solution for the Treatment of Cutaneous and Anogenital Warts: A Narrative Review

**DOI:** 10.3390/jcm15218592

**Published:** 2026-11-04

**Authors:** Giorgia Sbarra, Paolo Antonio Danza, Nicoletta Cassano, Gino Antonio Vena, Alexandre Raphael Meduri, Francesca Ambrogio, Giuseppe Demichele, Aurora De Marco, Domenico Bonamonte, Antonio Facciorusso, Giulia Ciccarese, Caterina Foti

**Affiliations:** 1 Section of Dermatology, Department of Precision and Regenerative Medicine and Jonian Area, University Aldo Moro of Bari, Piazza Giulio Cesare, 1, 70124 Bari, Italy; giorgiasbarra95@gmail.com (G.S.); a.meduri@phd.uniba.it (A.R.M.); dottambrogiofrancesca@gmail.com (F.A.); giuseppe.demichele08@gmail.com (G.D.); aurorademarco94@gmail.com (A.D.M.); domenico.bonamonte@uniba.it (D.B.); caterina.foti@uniba.it (C.F.); 2 Istituto Nazionale Previdenza Sociale (INPS), Lungomare Nazario Sauro, 41, 70124 Bari, Italy; dott.danza@libero.it; 3 Dermatology Private Practice Barletta, 76121 Barletta-Andria-Trani, 73100 Lecce, Italy; nicoletta.cassano@yahoo.com (N.C.); ginovena@gmail.com (G.A.V.); 4 Gastroenterology Unit, Department of Experimental Medicine, University of Salento, 73100 Lecce, Italy; antonio.facciorusso@unisalento.it

**Keywords:** cutaneous warts, genital warts, HPV, KOH, potassium hydroxide, recurrence, response, safety, treatment, viral warts

## Abstract

**Background**: Multiple options exist for the treatment of viral warts, but there is not yet a treatment capable of inducing a complete sustained response in all cases. The aim of this narrative review was to evaluate the efficacy and safety of KOH solution in cutaneous and anogenital warts. **Methods**: A literature search was conducted using the PubMed, Ovid Medline, Google Scholar, Scopus and Embase electronic databases and focusing on articles published in English that described the effects of monotherapy with KOH solution on different wart types. **Results**: We selected 24 studies involving 2083 patients, of whom 1423 were recruited in KOH groups. The heterogeneity in study designs, patient populations, outcomes and intervention characteristics (e.g., concentration of KOH, frequency of application, and treatment duration) precluded the possibilities of performing a meta-analysis and making direct comparisons among the studies. In many studies, there were small patient samples and short follow-up periods. However, despite such limitations, the available results regarding the use of KOH solution for warts seem to show efficacy rates comparable to those of established therapies, including TCA solution and cryotherapy. Side effects were generally described as mild and transient. KOH-related adverse events include burning sensation, erythema, dyspigmentation, and superficial erosions. **Conclusions**: Preliminary data suggest the potential clinical utility of KOH solution for the treatment of warts, although more data are needed to obtain definite conclusions and to optimize treatment protocols. Further research should include high-quality studies with extended follow-up periods and standardized outcome measures.

## 1. Introduction

Warts are common benign epithelial lesions secondary to human papillomavirus (HPV) infection that may develop on different body areas. The principal types include common, flat (plane), plantar, palmar, periungual, and genital/anogenital warts (condylomata acuminata/condyloma acuminata).

Cutaneous warts are caused by HPV types from all five genera and are common among adults and children. Common, plane and plantar warts are usually associated with HPV-2, -3, -10, -27 and -57 from the α-genus; HPV-4, -60 and -65 from the γ-genus; and HPV-1 and -63 from the μ-genus [1]. Cumulative exposure to cutaneous wart-associated HPVs is very high, although there are fewer epidemiologic data on such HPVs than on oncogenic and anogenital wart-associated HPVs. Four HPV types (HPV-1, HPV-2, HPV-27 and HPV-57) are believed to be responsible for more than 80% of cutaneous warts in the general population [2]. Low-risk HPV-6 and HPV-11 are implicated in most cases of anogenital warts, one of the most common sexually transmissible infections, with a prevalence of 0.2% to 5.1% worldwide and a peak incidence in the 20–29-year age group [1,2].

Spontaneous resolution of warts is possible but unpredictable, and precise data on the natural course are still scanty. In many cases, however, lesions tend to spread and become large and persistent, especially in severely immunocompromised patients, such as people with primary immunodeficiencies, people living with human immunodeficiency virus (HIV) infection and solid organ transplant recipients [3,4].

Notably, especially when the warts are located at genito-anal sites, the disease may impact the physical, psychological and sexual health of patients and negatively influence their quality of life [5].

Therefore, treatment is usually offered, and various therapeutic options are available. There is not yet a treatment capable of inducing a complete sustained response in all cases, and therapeutic management can be difficult [6,7,8].

The therapeutic armamentarium comprises a variety of treatments performed by the patients or their caregivers and physicians. Keratolytics and physical destructive treatments are among the traditional therapeutic options for cutaneous warts, with cryotherapy and topical salicylic acid as the most commonly used [9]. Current management strategies for external anogenital warts include patient-applied treatments (such as imiquimod, podophyllotoxin, and sinecatechins) and provider-administered therapies [such as cryotherapy, trichloroacetic acid (TCA), laser therapy, nitric-zinc complex solution, and surgical excision] [10,11,12,13]. Despite this broad therapeutic spectrum, no single intervention has emerged as universally superior. For instance, as concerns treatments for genital warts, cure rates range from 50% to 90%, and recurrence rates remain high, at 10% to 40% [13].

Potassium hydroxide (KOH) is a strong alkali with caustic properties that is licensed for the treatment of molluscum contagiosum, and its efficacy is attributed to the ability to dissolve keratin, causing an inflammatory reaction [14,15].

Various studies have evaluated topical KOH in the treatment of viral warts, but to the best of our knowledge, no comprehensive review has examined the efficacy and safety of KOH solution, used at different concentrations, for both cutaneous and anogenital warts. In addition, KOH monotherapy has not been mentioned in reviews or guidelines regarding cutaneous warts and has seldom been cited as a possible remedy for anogenital warts [16,17,18,19,20].

The objective of the present manuscript is to review the efficacy and safety of KOH solution in patients diagnosed with cutaneous and/or genital warts.

## 2. Materials and Methods

A literature search using the PubMed, Ovid Medline, Google Scholar, Scopus and Embase electronic databases was carried out, using the following relevant keywords: warts, wart, condyloma acuminata, condylomata acuminata, potassium hydroxide and KOH. Search reproducibility was guaranteed by providing full, unedited search strings for each consulted database (Supplementary Table S1). We evaluated articles published in English, available until 30th April 2026, that described the effects of monotherapy with KOH solution, regardless of concentration or duration of administration, on genital/anogenital and cutaneous warts in male and/or female patients of any age. Case reports and abstracts were excluded, as well as articles considering formulations different from aqueous solution (e.g., cream). Articles that did not specify the type of topical KOH formulation and those that examined the effect of KOH solution as a part of combination therapies were also excluded.

Articles were selected by two independent authors, and titles and abstracts were preliminarily reviewed for inclusion by the same authors. Duplicate articles were identified, and full-text articles were retrieved if preliminary review gave rise to doubts. Any discrepancies or disagreements between the two independent authors were resolved by involving a third author.

The authors independently extracted data from the selected articles, and the following items were collected: authors, country, year of publication, study period and design, clinical features (type of warts, number and affected sites), demographic characteristics of the participants, intervention details and assessed outcomes. When hands and feet were indicated as affected sites without specifying the exact localization, lesions were regarded as common warts.

We included studies that reported clinical response in terms of complete clearance (disappearance of all warts) or excellent improvement (reduction of more than 75% in wart number and/or size).

In order to align the results, the rate of responders was calculated using the total number of patients who completed the trial and were evaluated in the final analysis as the denominator. In fact, in the majority of studies examined in our review, study completers were included in the response analysis. Recurrence rates (calculated as the percentage of patients who had a complete response at the end of the treatment period) and safety data (frequency and type of adverse events) were also obtained. Efficacy data were extracted for the active comparator in controlled studies, and differences in the safety profile were also retrieved. Data on recurrence that were considered incomplete or doubtful (e.g., recurring lesions reported in patients with unknown response status) were not included.

Where data were ambiguous, study authors were contacted for additional information, though replies or clarifications were not always obtained.

A limitation of this study is the exclusion of non-English publications. However, given that the vast majority of high-impact research on warts/condylomas is indexed in major databases in English, it is unlikely that key clinical evidence was omitted.

## 3. Results

### 3.1. Details of the Included Studies

A total of 24 articles were selected (Table 1) [21,22,23,24,25,26,27,28,29,30,31,32,33,34,35,36,37,38,39,40,41,42,43,44].

The present review collected data of 2083 participants (1936 examined in the final analysis), of whom 1423 were recruited in KOH groups. Patients of any age were evaluated in most studies, whereas some authors examined only adults [32,33,34,35,38] or only children [28,29]. Existing evidence is derived mainly from Asian and African cohorts, revealing geographic disparities in research and restricting the generalizability of our findings. Three studies enrolled only men with genital warts [32,33,34], and another study enrolled only women with genital warts [36]. Different types of warts were treated: plane in eight studies [21,22,23,24,25,26,27,28], genital/anogenital in seven [32,33,34,35,36,37,38], palmar and/or plantar in two [29,30], only plantar in one study [31], common in three [39,41,43], and various types of non-genital warts in three studies [40,42,44]. All studies were prospective, and the majority were controlled [21,22,23,24,25,26,27,28,29,30,31,33,34,35,36,37,38,39,40,42,43,44]. Randomization was specified in 13 articles [22,25,28,29,30,31,33,34,35,38,39,42,44]. Intra-individual comparisons were made in four studies [26,31,33,36].

Most studies had small sample sizes and exhibited a moderate/high risk of bias (Supplementary Table S2).

In controlled trials, details regarding baseline differences between groups were frequently described [21,22,23,24,25,27,28,34,35,37,38,40,42,44], with most studies reporting no differences in terms of age [21,22,23,24,25,27,28,34,35,37,40,42,44] and gender [21,22,23,24,25,27,28,35,38,40,44] and fewer studies showing no differences in terms of number [21,22,23,24,25,34,35,37,42], duration [21,22,23,24,25,27,28,35,37,42] or size [25,34,42] of lesions. On the contrary, Hailu and Areda found more males (60%) in the group of patients treated with KOH than in the TCA group (26.2%) [37], whereas in the patient population analyzed by Kandil et al., there were statistically significant differences in the duration of disease between groups (longer in the KOH 10% group) [40]. Two uncontrolled trials evaluated KOH solution in a unique concentration [32,41]. Three studies assessed the effects of different concentrations of KOH [21,23,44], one study examined two different KOH concentrations and placebo (topical saline solution) [40], and another study compared two KOH concentrations with cryotherapy [39]. Active comparators were TCA in seven controlled trials [22,25,27,28,29,31,37] and cryotherapy in six studies [26,30,33,34,38,39], whereas single studies evaluated KOH versus tretinoin 0.05% cream [24], a 0.5% 5-fluorouracil and 10% salicylic acid combination (FU+SA) [35], carbon dioxide (CO)_2_ laser [36], and hydrogen peroxide 20% solution [43] and a four-arm trial examined intralesional vitamin D3, intralesional zinc sulfate 2% and placebo (normal saline) [42]. Characteristics of the studies (design, duration, eligibility criteria and outcomes), as well as treatment details (duration, KOH concentration, frequency and modality of application), were heterogeneous. Treatment duration was considered the period until clearance and up to the timepoint when the response was assessed, usually in the range of 4 to 12 weeks. KOH concentrations ranged from 5% to 40%, and the most frequently used concentrations were 5% and 10%. In the majority of cases, the solution was used once daily, whereas it was applied twice daily at a concentration of 5% in two studies involving patients with genital warts [33,36]. Weekly administration was sometimes applied for plane warts (with concentrations of 10% or 30%) [22,25,27,28] or for plantar warts using KOH 40% solution [31]. As concerns the application of KOH, many studies specified that it was performed by the patients or their caregivers [21,23,24,29,30,32,33,34,35,36,37,38,39,40,41,44], and fewer studies specified that KOH was applied by the physician with weekly administration [22,25,28,31], whereas other articles did not specify precisely who applied the solution. Nevertheless, its daily use, especially at night or in the evening, suggested home treatment.

Many authors reported application using a wooden stick or a cotton-tip applicator [21,22,23,24,25,27,31,32,34,35,36,37,38,39,42,44], and some authors specified that the perilesional area was covered with a layer of Vaseline/petroleum jelly to prevent irritation [22,25,27,28,35,41,42,44].

Fourteen studies reported the use of photos to support the assessment of clinical outcomes [21,23,24,26,27,28,31,33,35,39,40,41,42,43]. Dermoscopy was employed to aid in diagnosis and/or monitor treatment effects on rare occasions [31,42]. Table 2 and Table 3 synthesize only the complete response rates at the end of treatment; outcomes assessed differently (e.g., >75% improvement soon after treatment or complete clearance after a treatment-free follow-up period) are described narratively.

As previously stated, in most studies, outcomes were assessed using study completers and not the enrolled patients. In various trials, the number of enrolled patients corresponded to the number of patients analyzed for response (Table 1). Reasons for discontinuation or exclusion after enrollment were specified in some publications, and patients excluded from the analysis were often described as follows: defaulters/defaulted for unknown reasons [21,23,24,38], lost to follow-up [22,32,33,34] or non-compliant [36,39]. Moreover, one patient withdrew from a study because of an adverse event [22], one patient was not included in the analysis of another study after the development of a urethral lesion [32] and another was excluded after the detection of hepatitis C virus seropositivity [33]. Only rarely were subjects who were lost to follow-up considered treatment failures [34].

As concerns recurrence of warts in the follow-up post-treatment period, a precise definition was not found in the majority of the cited studies.

### 3.2. Clinical Response to KOH

Many studies showed the efficacy of KOH in terms of complete clearance of warts at the end of the treatment period. Efficacy data of these studies are reported in Table 2.

Conversely, in a minority of studies, the outcome was assessed in a different way [29,31,32,34,35,43]—namely, considering an improvement greater than 75% at the end of the treatment period or taking into account the total clearance after a treatment-free follow-up period. In particular, in male patients with genital warts, Loureiro et al. used KOH 5% once daily for up to 2 months [32], whereas Camargo et al. instructed patients to apply KOH 5% daily for up to 3 months, evaluating the clinical response at a follow-up post-treatment visit [34]. Patients who were completely wart-free without recurrences one month after treatment completion represented 87.5% of the treated patients in the former study [32] and 65% in the latter study [34]. Hanif et al. assessed the clinical outcome 8 weeks after treatment and observed complete sustained clearance in 77% of patients who applied KOH 10% solution once daily on palmar and/or plantar warts for 8 weeks [29]. An excellent response (>75% reduction) was noted after 12 weeks in 70% and 88.7% of patients with genital/anogenital warts by Işik et al. [35] and Javame Ghazvini et al. [38], respectively, using KOH 5% solution once daily, and in 30% of patients with common warts treated with KOH 15% solution once daily and examined at week 4 by Hassan et al. [43]. Farouk et al. found that KOH 40% solution applied once weekly by the physician on plantar warts produced an improvement greater than 75% in 27.6% of patients after 6 weeks of treatment and in 65.2% at a follow-up visit 2 months after treatment [31].

Regarding the efficacy in terms of complete response assessed at the end of a treatment period ranging from 4 weeks to 12 weeks (Table 2), this outcome was achieved with the application of KOH 5% solution once daily in 56.7% to 80.4% of patients with plane warts [21,24,26], in 70.5% of patients with genital warts [38], and in 69.2% of patients with common warts [39], whereas the disappearance of genital warts was documented in 84.6–88.9% of patients in whom the solution was applied at the same concentration twice daily [33,36]. A single daily application of KOH 10% solution was used in various studies involving different patient populations and caused a complete response in more than half of the patients treated as follows: 81–82% in plane warts [21,23], 73.3% in palmoplantar warts [30], 87.5% in anogenital warts [37], and 54.3–84.6% in common warts [39,41]; in contrast, studies evaluating different wart variants (more frequently common, plane, and plantar types) reported rates of 55% to 75% [40,42,44]. Complete response was detected in 87.5% of patients with plane warts [23] and 80% of subjects with various wart types [40] using KOH 15% solution once daily and in 65.6% of subjects with common, plantar or plane warts using KOH 20% once daily [44].

In a few studies examining the effect after 12 weeks of once weekly administration for plane warts, 26% [22] to 75% [25] of patients using KOH 10% were completely cured, while 23.3% [27] to 31.8% [28] using a concentration of 30% were completely cured (Table 2).

As concerns the effects of different KOH concentrations, some authors found no difference in the efficacy for plane warts between groups treated with 5% and 10% [21] or between those treated with 10% and 15% [23], with better response observed for smaller lesions and a faster action with higher concentrations [21,23]. On the contrary, the results obtained by Wickremasinghe and Karunasekara showed the superiority of KOH 10% over KOH 5% for the treatment of common warts [39]. Elgayar et al. noted better responses with KOH 10% in plane warts as compared to common and plantar warts [42].

### 3.3. Clinical Response to KOH Versus Other Treatments

Table 3 summarizes the rate of complete clearance at the end of treatment in controlled studies.

In controlled trials, no relevant differences in the achievement of complete response were detected between the KOH groups and patients who received TCA at various concentrations and cryotherapy in many studies [22,25,26,27,28,30,31,34,37,38,39], and single studies reported the comparable efficacy of KOH 5% and tretinoin 0.05% cream at the end of the trial for plane warts [24] and CO_2_ laser for anogenital warts [36].

A few controlled studies defined the best response grade as regression greater than 75% [35,43] and showed no significant differences between KOH 5% and FU+SA in the treatment of anogenital warts (70% versus 76.7% at week 12) [35] and between KOH 15% and hydrogen peroxide 20% for common warts (30% versus 48% at week 4) [43].

In some articles, similar efficacy results with KOH and the active comparators were specified to involve both response at the end of treatment and recurrence [36,37,38,39,43].

Instead, some publications highlighted the presence of differences between treatments. A few authors described superior clinical outcomes in patients treated with KOH compared to other therapeutic modalities: KOH 10% once daily versus 35% TCA once weekly for palmar and/or plantar warts [29] and KOH 5% twice a day versus cryotherapy for genital warts [33]. Jayaprasad et al. evaluated the effects of once weekly application of KOH 10% and TCA 30% in plane warts and found statistically significant better results in favor of KOH for warts sized 1–5 mm, with 2 to 9 months duration and numbers of 1 to 10, as well as in terms of subjective response (patient’s perception of improvement) [22]. As concerns KOH 5%, a shorter duration of treatment as compared to cryotherapy was occasionally observed in plane warts [26] and genital warts [34], whereas a greater efficacy as compared to tretinoin cream in plane warts was reported at week 4, with similar efficacy results present in the two treatment arms at the end of the treatment phase (4 weeks for KOH and 8 weeks for tretinoin) [24]. The healing time was reported to be shorter with KOH 10% applied once daily as compared to TCA 80% used once weekly for anogenital lesions [37]. The results obtained in plantar warts with KOH 40% and TCA 40%, both applied once weekly, showed that dermoscopic signs of complete clearance without recurrence during the follow-up period were more frequent with KOH (88.6% versus 66.7%) [31].

Only two studies were placebo-controlled [40,42], enrolling a total of 40 patients in the placebo groups (20 for each trial). In the former study [40], there were statistically significant differences between KOH and placebo (saline solution) (*p* < 0.001) with respect to complete response at the end of 8 weeks of treatment, as this outcome was observed in 75% of patients treated with KOH 10% once daily, 80% of patients treated with KOH 15% once daily, and 10% of subjects in the placebo group. In the latter study [42], after 6 weeks of treatment, complete responders represented 55% of patients treated with KOH 10% once daily and 10% of patients who received intralesional 0.9% normal saline every 2 weeks.

### 3.4. Recurrences

Precise data regarding the recurrence rate were available only for some studies where the frequency was clearly defined as the percentage of patients who achieved complete response after the treatment period. In fact, in other studies, frequencies of recurrences appeared to be extrapolated from patients with unknown response status (e.g., patients with partial or total response or from the total treated patients irrespective of the response status).

In trials assessing the recurrence in complete responders with plane warts, lesions recurred in 5.8–19% of patients 3 months after treatment with KOH 5% once daily [21,24] and in 5.1% of subjects 3 months after treatment with KOH 10% once daily [21]. No recurrence was detected after 8 weeks from the end of treatment of genital warts with KOH 5% twice daily [33], at 3 months [40] or 6 months [42] after treatment of different non-genital wart types with KOH 10% solution once daily, at 3 months after treatment of non-genital warts with KOH 15% once daily [40] and 2–3 months after treatment with KOH 10% once weekly in patients with plane warts [22,25].

In controlled trials, most authors found no apparent differences in the recurrence rates between topical KOH and other active treatments. Similar numbers of recurrences were revealed in the follow-up post-treatment phase in some studies involving patients with plane warts (KOH 5% once daily versus topical tretinoin [24], KOH 5% once daily versus cryotherapy [26], KOH 30% once weekly versus TCA 30% weekly [27], and KOH 10% once weekly versus TCA 50% once weekly [25]) and in one study performed in patients with genital warts treated with KOH 5% solution or cryotherapy [33]. Other comparative studies clearly declared the absence of statistically significant differences in recurrences between KOH and other treatments in patients with plane warts (KOH 30% once weekly versus TCA 30% once weekly [28] and KOH 10% once weekly versus TCA 30% once weekly [22]) and genital/anogenital warts (KOH 5% twice daily versus CO_2_ laser therapy [36], KOH 10% once daily versus TCA 80% once weekly [37], KOH 5% once daily versus FU+SA once daily [35], and KOH 5% once daily versus cryotherapy [38]).

### 3.5. Tolerability

Table 4 shows a brief description of the type of side effects in the main trials carried out in patients with genital and cutaneous warts, and Table 5 summarizes the comparison of safety aspects between KOH solution and other treatments in controlled studies.

In most publications, the frequency of adverse events was calculated using the patients who completed the study and were included in the final analysis as the denominator. Instead, the proportion of patients with adverse events was based on the total enrolled population in the study by Javame Ghazvini et al. [38]. The denominator used to calculate the adverse event rates was not clearly defined in the studies by Camargo et al. [34] and Loureiro et al. [32], and the exact modality of assessment of side effects was not explicitly described in the study by Kalfa et al. [44].

Treatment with KOH 5% once daily was associated with a frequency of adverse events of 77.6–83.3% in patients with plane warts [21,24] and 100% in men with genital warts [32], whereas two daily applications of KOH 5% on genital warts caused adverse events in 24% of women [36] and 100% of male patients [33]. In three different trials, adverse events during the daily use of KOH 10% occurred in 30% of patients with diverse types of non-genital warts [40], 90.5% of patients with facial plane warts [21], and 73.8% of subjects with plane warts in various sites [23]. Ninety per cent of subjects with plane warts [23] and 40% of patients with various non-genital variants [40] experienced adverse events when treated with KOH 15% once daily. A weekly application of KOH 30% on plane warts caused adverse events in 100% of cases [27,28]. Side effects were recorded in 24.2% of patients with plantar warts receiving KOH 40% once weekly [31].

As concerns the tolerability profile of different KOH concentrations, Al-Hamdi and Al-Rahmani noted that side effects in facial plane warts were more common with KOH 10% as compared to KOH 5% (*p* = 0.0001) [21]. Similarly, Wickremasinghe and Karunasekara reported higher rates of erythema and pain with KOH 10% than KOH 5% (*p*-values not reported) [39], and Rezk and Gaber found a higher frequency of side effects in plane warts on various sites with KOH 15% than with KOH 10% (*p* = 0.05) [23]. A trend towards more side effects was observed by Kalfa and Elashibi with KOH 20% as compared to KOH 10% in the treatment of various wart types [44]. Conversely, Kandil et al., examining patients with non-genital warts of various types, found no statistically significant differences between KOH groups (10% and 15%) in side effects [40].

Discontinuations due to adverse events were rarely reported during treatment with topical KOH and occurred in two patients with plane warts treated with KOH 10% once weekly who developed irritant contact dermatitis [22].

In many publications, adverse events were defined as tolerable, mild and transient. Almost all studies reported the development of local symptoms (described as itching or burning sensation and more rarely as pain or stinging) with variable frequency. Other adverse events mentioned in the majority of studies were erythema and pigmentary changes (recorded as dyspigmentation, hypo-depigmentation or hyperpigmentation). In general, the frequency of side effects varied considerably across studies. Erosions and/or ulcerations occurred less commonly than the above-mentioned adverse events, but they were more frequently associated with treatment of genital warts. Scarring was rarely reported.

As concerns comparative studies in patients with plane warts, KOH 5% appeared to cause more side effects than topical tretinoin [24] and to less frequently cause scarring or a crust than cryotherapy [26]; treatment with KOH 10% once weekly was found to be associated with fewer side effects than TCA 30% [22] or 50% [25] once weekly, whereas there were no differences in tolerability between KOH 30% once weekly and TCA 30% once weekly [27,28], except for hypopigmentation, which was more commonly observed with KOH in one study [27].

For the treatment of genital/anogenital warts, the severity of pain was higher with TCA 80% once weekly than with KOH 10% solution once daily [37], mixed results were obtained with KOH 5% once or twice daily in comparison with cryotherapy [33,34,38], and a similar safety profile was shown for KOH 5% once or twice daily when compared to both FU+SA and CO_2_ laser [35,36]. No significant differences in side effects were revealed between KOH 40% once weekly and TCA 40% once weekly in patients with plantar warts [31] or between KOH 15% and hydrogen peroxide 20% used once daily for common warts [43], whereas among the patients with common warts examined by Wickremasinghe and Karunasekara, subjects treated with cryotherapy complained of pain more frequently than those treated with KOH 5% or 10% [39].

## 4. Discussion

Our narrative review was carried out with the aim of analyzing the efficacy and safety of KOH solution used at different concentrations in cutaneous and anogenital warts, trying to summarize the data available in the current literature. The heterogeneity in study designs, patient populations, outcomes (e.g., definition of response and time of assessment of response and recurrence) and intervention characteristics (e.g., for KOH, concentration, frequency of application, and treatment duration, as well as active comparators in controlled trials) precluded the possibility of performing a meta-analysis and making direct comparisons of the efficacy and safety profiles among the studies included in the present review. Intra-individual comparisons, which can provide more robust results by removing patient-related confounding factors, were performed in very few studies [26,31,33,36]. Various studies appeared to have methodological issues, especially with regard to the definition and reporting of response and recurrence. It should be pointed out that the assessment of outcomes was generally made without an intent-to-treat approach, and the response rates were frequently calculated using study completers. This could have led to an overestimation of efficacy.

Nevertheless, despite such obvious limitations, the available evidence suggests that KOH can be considered a viable option for the treatment of cutaneous and genital warts. The results of most comparative studies seem to show the non-inferiority of KOH solution, with efficacy rates comparable to those of established therapies, including TCA solution and cryotherapy, which were the active comparators most frequently investigated.

Potential advantages of topical KOH for both patients and the healthcare system include the possibility of patient self-application, ease of use, inexpensiveness, and wide availability, which can be particularly important in resource-limited settings [13,45]. Moreover, a relatively fast onset of action and short duration of treatment were sometimes underscored, especially for smaller lesions.

Abalos-Babaran et al. published a systematic review that included four low-quality randomized controlled studies (197 patients) [45], examining the effect of KOH 5% for anogenital warts [33,34,35,36]. Their results showed that patients treated with KOH had a higher clearance rate compared to cryotherapy, but the difference did not reach statistical significance, and there were no significant differences among patients treated with KOH, FU+SA and CO_2_ laser vaporization in terms of mean lesion count or size.

These findings were corroborated by another article [13] that analyzed five comparative studies involving 351 patients with condyloma acuminata treated with topical KOH [34,35,36,37,38], which was shown to induce complete response rates comparable to those of other treatments, including cryotherapy, TCA, and CO_2_ laser therapy.

The results of the studies included in our review seem to confirm the conclusions drawn from the above-cited systematic reviews regarding the effectiveness of topical KOH in anogenital warts and support similar findings for cutaneous warts.

The safety profile of KOH was generally favorable, with common drug-related adverse events being burning sensation, erythema, dyspigmentation, and superficial erosions. These events can be considered a natural and predictable consequence of the mechanism of action of KOH, like other treatments with caustic and proinflammatory effects. However, in most publications, the rate of adverse events was based on the patients who completed the study and were included in the final analysis. This could have caused an underestimation of the actual prevalence of side effects in the treated patients. Notably, serious or systemic adverse events were not recorded in any studies, and discontinuation due to side effects occurred very rarely. Moreover, many authors pointed out that local adverse events caused by KOH were mild and transient, tolerable and manageable.

Studies exploring the clinical activity of different concentrations of KOH solution mostly showed a dose-dependent relationship between the safety profile and the concentration, with a trend towards higher rates of side effects with increasing concentrations of KOH. Moreover, the outcomes resulting from the use of a topical caustic agent may be influenced by multiple factors, and in this type of treatment, accurate patient selection and monitoring are of paramount importance, as well as the implementation of measures aimed at improving the risk/benefit ratio and patient compliance, in addition to limiting tolerability issues. The optimization of treatment protocols is also required, and more data are needed to define the optimal KOH concentration, treatment duration and modalities of application according to the clinical characteristics of warts.

Further research should include high-quality studies with extended follow-up periods and standardized outcome measures. The role of KOH in combination therapies for warts is another interesting aspect that deserves attention in future investigations.

## Figures and Tables

**Table 1:** Potassium hydroxide solution for the treatment of warts: General characteristics of the studies included in the present review.

First Author (Year), Country [Reference]	Study Type	Study Period	Patients			Warts		
			Total Enrolled (Analyzed *) N	% Males	Age (Years) Range or Other Data if Range NR	Type	Affected Sites	N Range or Other Data if Range NR
Al-Hamdi (2012), Iraq [21]	P, OL, C 2 arms: KOH 5%, KOH 10%	March 2008–October 2009	250 (202)	46.5	3–45	Plane	Face	1–30
Jayaprasad (2016), India [22]	P, R, OL, C 2 arms: KOH 10%, TCA 30%	October 2013–March 2015	60 (54)	57.4	6–78	Plane	Various (face in 66.7%)	3–50
Rezk (2017), Egypt [23]	P, OL, C 2 arms: KOH 10%, KOH 15%	NR	100 (82)	47.6	4–68	Plane	Face, hands and legs	1–9
Al-Akkam (2018), Iraq [24]	P, OL, C 2 arms: KOH 5%, tretinoin 0.05% cream	April 2015–July 2016	66 (58)	31	3–40	Plane	Face	5–70
Yaghoobi (2019), Iran [25]	P, R, C ^ 2 arms: KOH 10%, TCA 50%	August 2017–February 2018	64	45.3	4–51	Plane	Various (head and neck in about two thirds of patients)	3–30
Galal (2020), Egypt [26]	P, OL, C Intra-individual comparison 2 arms: KOH 5%, Cryotherapy	26 December 2018–1 December 2019	30	40	5–32	Plane	NR	NR
Alawady (2020), Egypt [27]	P, C 2 arms: KOH 30%, TCA 30%	November 2019–March 2020	60	56.7	Mean 14.4–15.6; median 11–13.5	Plane	Various (head in 76.6% of cases)	NR
Alawady (2022), Egypt [28]	P, SB, R, C 2 arms: KOH 30%, TCA 30%	October 2019–March 2020	45	66.7	7–18 (mean 11.3)	Plane	Face in 82.2% of cases, also neck and upper limbs	NR
Hanif (2022), Pakistan [29]	P, R, C 2 arms: KOH 10%, TCA 35%	13 November 2016–12 May 2017	148	49.3	3–12	Palmar and/or plantar	-	NR
Bashir (2024), Pakistan [30]	P, R, C 2 arms: KOH 10%, cryotherapy	NR (6-month period)	120	38.3	15–60 (mean 22.9)	Palmoplantar	-	NR (up to 2 warts in 66.7% of cases)
Farouk (2025), Egypt [31]	P, SB, R, C Intra-individual comparison 2 arms: KOH 40%, TCA 40%	December 2022–July 2023	33	36.4	14–50	Plantar	-	2–4
Loureiro (2008), Brazil [32]	P, OL KOH 5%	NR	35 (32)	100	18–49 (mean 29.3)	Genital	-	1–15
Yaghoobi (2014), Iran [33]	P, R, C Intra-individual comparison (right vs left) 2 arms: KOH 5%, cryotherapy	March 2009–March 2010	36 (26)	100	18–60 (mean 35)	Genital	-	NR
Camargo (2014), Brazil [34]	P, OL, R, C Random allocation on an alternative basis 2 arms: KOH 5%, cryotherapy	February 2009– December 2009	48 (42)	100	18–74 (mean 31.1)	Genital	Glans, foreskin, penile shaft, and pubic and scrotal area	1–20
Işik (2014), Turkey [35]	P, R, OL, C 2 arms: KOH 5%, FU + SA	NR	60	68.3	NR (>18 years; mean 37 in one group, 33.7 in the other)	Anogenital	-	NR (mean 17.0 in one group and 16.1 in the other)
Asadi (2016), Iran [36]	P, OL, C Intra-individual comparison (right versus left) 2 arms: KOH 5%, CO_2_ laser	March 2014–August 2014	70 (63)	0	15–55	Genital	External genital areas (including perianal); the majority on the labia major	1–40
Hailu (2021), Ethiopia [37]	P, OL, Non-R, C 2 arms: KOH 10%, TCA 80%	March 2017–May 2018	90 (82)	42.7	4–52 (mean 28.4)	Anogenital	-	NR (mean 10.1)
Javame Ghazvini (2025), Iran [38]	P, R, C 2 arms: KOH 5%, cryotherapy	2022–2023	98 (91)	43.9	23–36 (mean 28.4)	Genital	External genital areas (including perianal)	3–8.5
Wickremasinghe (2010), Sri Lanka [39]	P, R, C 3 arms: KOH 5%, KOH 10%, cryotherapy	NR	300 (274)	49.3	3–60	Common	Various (facial and genital excluded)	1–30
Kandil (2016), Egypt [40]	P, non-R, C 3 arms: KOH 10%, KOH 15%, placebo (topical saline solution)	May 2014–September 2014	60	46.7	4–60	Nongenital: more commonly, plantar, and plane; less frequently periungual and palmar	Various (details NR)	NR
Makki (2022), Egypt [41]	P, OL KOH 10%	October 2020–January 2022	70	25.7	5–45 (mean 27.0)	Common	Dorsum of hands (48.6%), soles (44.3%), face (7.1%)	NR (mean 2.4)
Elgayar (2025), Egypt [42]	P, SB, R, C 4 arms: KOH 10%, intralesional vitamin D_3_, intralesional zinc sulfate 2%, placebo (0.9% normal saline)	Unclear (discordant information)	80	NR	13–60	Recalcitrant cutaneous warts (predominantly common and plantar, less frequently plane)	Predominantly acral sites	1–25
Hassan (2024), Egypt [43]	P, C 2 arms: KOH 15%, hydrogen peroxide 20% solution	NR	100	NR	NR	Common	NR	NR (mean 1.6)
Kalfa (2024), Libya [44]	P, R, C 2 arms: KOH 10%, KOH 20%	February 2022–January 2023	60	43.3	3–21 (mean 11.8)	Plantar (36.7%), common (35%), plane (28.3%)	Face, feet, hands, scalp	NR

C: comparative; FU+SA: 0.5% 5-fluorouracil and 10% salicylic acid combination; KOH: potassium hydroxide; N: number; NR: not reported; OL: open-label; P: prospective; R: randomized; SB: single-blinded; TCA: trichloroacetic acid. * Analyzed = patients who completed the study and were included in the final analysis (when the article specified a number different from the number of total enrolled patients); ^ blinding details not clearly specified.

**Table 2:** Complete response at the end of treatment with KOH solution in patients with warts.

First Author (Year)	Patients Treated with KOH		Warts		Complete Response (100% Clearance)
	Total Enrolled (Analyzed *) N % Males, if Reported	Age (Years) Mean and/or Range	Type	Mean N	
**5% once daily**					
Al-Hamdi (2012) [21]	125 (107)	13.1	Plane	7.0	*4 weeks*
	42.9%	3–45			80.4%
Al-Akkam (2018) [24]	33 (30)	15.5	Plane	22.2	*4 weeks*
	33.3%	5–40			70%
Galal (2020) [26]	30		Plane	NR	*4 weeks*
	40%	5–32			56.7%
Javame Ghazvini (2025) [38]	49 (44)	30	Genital	NR	*12 weeks*
		25–36			70.5%
Wickremasinghe (2010) [39]	100 (91)	NR	Common	NR	*8 weeks*
					69.2%
**5% twice daily**					
Yaghoobi (2014) [33]	36 (26)	35	Genital	NR	*8 weeks*
	100%	18–60			84.6%
Asadi (2016) [36]	70 (63)	28.6	Genital	12.5	*9 weeks*
	0%	15–50			88.9%
**10% once daily**					
Al-Hamdi (2012) [21]	125 (95)	13.7	Plane	6.75	*4 weeks*
	50.5%	4–43			82.1%
Rezk (2017) [23]	50 (42)		Plane	2.9	*4 weeks*
	33.3%	4–22			81%
Bashir (2024) [30]	60	NR	Palmoplantar	NR	*4 weeks*
	43.3%				73.3%
Hailu (2021) [37]	NR (40)	28.9	Anogenital	9.4	*12 weeks*
					87.5%
Wickremasinghe (2010) [39]	100 (91)	NR	Common	NR	*8 weeks*
					84.6%
Kandil (2016) [40]	20	24.25	Common, plane, plantar, palmar, periungual	NR	*8 weeks*
	45%	4–59			75%
Makki (2022) [41]	70	27.01	Common	2.4	*12 weeks*
	25.7%	5–45			54.3%
Elgayar (2025) [42]	20	26.3	Common, plantar, plane	NR (median 1)	*6 weeks*
		10–50			55%
Kalfa (2024) [44]	28	11.8	Common, plantar, plane	NR	*4 weeks*
		3–21			67.9%
**15% once daily**					
Rezk (2017) [23]	50 (40)		Plane	3.3	*4 weeks*
	62.5%	6–68			87.5%
Kandil (2016) [40]	20	30.8	Common, plane, plantar, palmar, periungual	NR	*8 weeks*
	45%	8–60			80%
**20% once daily**					
Kalfa (2024) [44]	32	11.8	Common, plantar, plane	NR	*4 weeks*
		3–21			65.6%
**10% once weekly**					
Jayaprasad (2016) [22]	30 (27)	28.9	Plane	16.2	*12 weeks*
	55.55%				25.9%
Yaghoobi (2019) [25]	32	19.25	Plane	10.9	*12 weeks*
	46.9%				75%
**30% once weekly**					
Alawady (2020) [27]	30	14.3	Plane	NR	*12 weeks*
	50%				23.3%
Alawady (2022) [28]	22	10.4	Plane	NR	*12 weeks*
	59.1%	7–18			31.8%

KOH: potassium hydroxide; N: number; NR: not reported. * Analyzed = patients who completed the study and were included in the final analysis (when the article specified a number different from the number of total enrolled patients).

**Table 3:** Complete response at the end of the treatment period in controlled studies comparing KOH with other treatments for warts.

First Author (Year) Type of Warts	Treated Patients (Analyzed *) N		Complete Response (100% Clearance)	
	KOH	Active Comparator	KOH (Max Treatment Duration/Rate)	Active Comparator (Max Treatment Duration/Rate)
**5% KOH once daily**				
Al-Akkam (2018) [24] Plane	33 (30)	33 (28)	*4 weeks* 70%	*8 weeks* 60.7% Tretinoin 0.05% cream once daily
Galal (2020) [26] Plane	30	30	*4 weeks* 56.7%	*4 weeks* 53.3% Cryotherapy every 2 weeks
Javame Ghazvini (2025) [38] Genital	49 (44)	49 (47)	*12 weeks* 70.5%	*12 weeks* 72.3% Cryotherapy (two 5–20 s freeze–thaw cycles) ^
Wickremasinghe (2010) [39] Common	100 (91)	100 (92)	*8 weeks* 69.2%	*8 weeks* 76.1% Cryotherapy with liquid nitrogen ^
**5% KOH twice daily**				
Yaghoobi (2014) [33] Genital	36 (26)	36 (26)	*8 weeks* 84.6%	*8 weeks* 50% Cryotherapy with liquid nitrogen ^
Asadi (2016) [36] Genital	70 (63)	70 (63)	*9 weeks* 88.9%	*9 weeks* 88.9% CO_2_ laser every 3 weeks
**10% KOH once daily**				
Bashir (2024) [30] Palmoplantar	60	60	*4 weeks* 73.3%	*4 weeks* 60% Cryotherapy with liquid nitrogen once weekly
Hailu (2021) [37] Anogenital	NR (40) §	NR (42) §	*12 weeks* 87.5%	*12 weeks* 78.6% TCA 80% weekly
Wickremasinghe (2010) [39] Common	100 (91)	100 (92)	*8 weeks* 84.6%	*8 weeks* 84.6% Cryotherapy with liquid nitrogen ^
Elgayar (2025) [42] Common, plantar, plane	20	20	*6 weeks* 55%	*6 weeks* 75% Intralesional vitamin D_3_ (every 2 weeks for 4 cycles)
		20		70% Intralesional zinc sulfate 2% (every 2 weeks for 4 cycles)
**10% KOH once weekly**				
Jayaprasad (2016) [22] Plane	30 (27)	30 (27)	*12 weeks* 25.92%	*12 weeks* 11% TCA 30% solution once weekly
Yaghoobi (2019) [25] Plane	32	32	*12 weeks* 75%	*12 weeks* 87.5% TCA 50% solution once weekly
**30% KOH once weekly**				
Alawady (2020) [27] Plane	30	30	*12 weeks* 23.3%	*12 weeks* 23.3% TCA 30% solution once weekly
Alawady (2022) [28] Plane	22	23	*12 weeks* 31.8%	*12 weeks* 26.1% TCA 30% solution once weekly

KOH: potassium hydroxide; TCA: trichloroacetic acid. * Analyzed = patients who completed the study and were included in the final analysis (when the article specified a number different from the number of total enrolled patients); ^ interval not specified or not clearly defined; § total number of enrolled patients: 90 (82 analyzed).

**Table 4:** Type of adverse events associated with the use of KOH solution at different concentrations for the treatment of warts.

First Author (Year)	Wart Types	Concentration of KOH and Treatment Details (Max Duration)	Type of Side Effects and Corresponding Frequency ^	Relevant Notes
Al -Hamdi (2012) [21]	Plane (face)	5% Once daily, at night (4 weeks)	Itching, 39.3%; burning sensation, 27.1%; erythema, 23.3%; dyspigmentation, 6.54%	
Al-Akkam (2018) [24]	Plane (face)	5% Once daily, at night (4 weeks)	Burning sensation, 56.7%; itching, 36.7%; dyspigmentation, 33.3% (most frequently)	
Galal (2020) [26]	Plane	5% Once daily, at night (4 weeks)	Erythema, 53.3%; itching, 50%; hypopigmentation, 33.3%; hyperpigmentation, 23.3%; crust, 6.7%; scar, 3.3%	
Loureiro (2008) [32]	Genital in males	5% Once daily (8 weeks)	Mild inflammation (erythema and edema), 100%; superficial erosions, 56%; stinging, 22%; hypopigmentation, 9%	
Yaghoobi (2014) [33]	Genital in males	5% Twice a day (8 weeks)	Burning, 100%; ulceration, 90% §	No secondary infection No discontinuations due to complications
Camargo (2014) [34]	Genital in males	5% Daily (3 months)	Superficial erosions, 50%; mild pain or stinging, 16.7%; residual hypopigmentation, 16.7%	
Işik (2014) [35]	Anogenital	5% Once daily, in the evening (12 weeks) perilesional Vaseline	Mild and transient erythema and burning sensation, 60%	Mild transient burning sensation and erosion reported as the most common adverse events Numerical data NR
Asadi (2016) [36]	Genital in females	5% Twice a day (9 weeks)	Erosion, 14.28%; burn, 11.11%; erythema, 6.34%; hyperpigmentation, 3.17%	
Javame Ghazvini (2025) [38]	Genital	5% Once daily (12 weeks)	Burning, 14.3%; hyperpigmentation, 12.2%; hypopigmentation, 8.2%; erosion, 6.1%; erythema, 4.1%	
Wickremasinghe (2010) [39]	Common (various areas; facial and genital excluded)	5% Once daily (8 weeks)	Pain, 17.5%; erythema, 14.2%	
Al-Hamdi (2012) [21]	Plane (face)	10% Once daily, at night (4 weeks)	Burning, sensation; 38.9%; dyspigmentation, 28.4%; erythema, 28.4%; itching, 15.8%	
Jayaprasad (2016) [22]	Plane (face in 67% of patients)	10% Once weekly (12 weeks) perilesional Vaseline	Transient burning or itching, 25.92%; hypopigmentation, 22.2%; transient erythema, 11.11%.	No scarring Irritant contact dermatitis responsible for treatment discontinuation in two cases
Rezk (2017) [23]	Plane (face, hands and legs)	10% Once daily, at night (4 weeks)	Itching, 35.7%; burning sensation, 19.0%; erythema, 14.3%; dyspigmentation, 4.8%	
Yaghoobi (2019) [25]	Plane (head and neck in about two thirds of patients)	10% Once weekly (12 weeks) perilesional Vaseline	Transient burning sensation, 78.1%; dyspigmentation, 43.7%; crusting, 31.2%	
Hailu (2021) [37]	Anogenital	10% Once daily (12 weeks)	Ulceration, 12.5%	
Wickremasinghe (2010) [39]	Common (various areas, facial and genital excluded)	10% Once daily (8 weeks)	Pain, 67.3%; erythema, 46.1%; hypopigmentation, 1%	
Kandil (2016) [40]	Common, plane, plantar, palmar, periungual	10% Once daily, at night (8 weeks)	Itching, burning sensation, erythema, and temporary dyspigmentation, 30%	Rate for each event NR Adverse events described as tolerable and transient
Makki (2022) [41]	Common (dorsum of hands, plantar, face)	10% Once daily, at night (12 weeks) perilesional Vaseline	Post-inflammatory hyperpigmentation, 31.4% (more common on the dorsum of the hands)	Tolerable and transient adverse effects (e.g., burning sensation, erythema, stinging, and erosion) were mentioned (precise data NR)
Elgayar (2025) [42]	Recalcitrant warts (common, plantar, less frequently plane)	10% Once daily, at night (6 weeks) perilesional Vaseline	Transient edema and erythema, 50%; transient depigmentation, 20%	
Kalfa (2024) [44]	Plane, common, plantar (more frequently feet, face, and hands)	10% Once daily, at night (4 weeks) perilesional petroleum jelly	Erythema, 7.14%; itching, 3.6% (4 weeks)	
Rezk (2017) [23]	Plane (face, hands and legs)	15% Once daily, at night (4 weeks)	Burning sensation, 35%; dyspigmentation, 25%; itching, 17.5%; erythema, 12.5%	
Alawady (2020) [27]	Plane (face in most cases)	30% Once weekly (12 weeks) perilesional Vaseline	Transient erythema, 100%; mild burning sensation, 100%; hypopigmentation, 63.3%; hyperpigmentation, 6.7%	
Alawady (2022) [28]	Plane (predominantly the face)	30% Once weekly (12 weeks) perilesional Vaseline	Transient mild erythema, 100; transient mild burning sensation, 100%; hypopigmentation, 63.6%; scarring, 27.3%; hyperpigmentation, 9.1%	
Farouk (2025) [31]	Plantar	40% Once weekly (6 weeks)	Black eschar, 12.1%; burning sensation, 6.1%; ulcer, 3%; subcorneal hemorrhage, 3%	Adverse events described as minimal
Kandil (2016) [40]	Nongenital (common, plane, plantar, palmar, periungual)	15% Once daily, at night (8 weeks)	Itching, burning sensation, erythema, and temporary dyspigmentation, 40%	Rate for each event NR Adverse events described as tolerable and transient
Hassan (2024) [43]	Common (not specified)	15% Once daily (4 weeks)	Pain, 30%; hyperpigmentation, 10%	Infection and Koebner’s phenomenon occurred rarely Complications described as minor Precise data NR and not clearly inferable from the graphic
Kalfa (2024) [44]	Plane, common, plantar (feet 46.7%, face 26.7%, hands 21.6%, scalp 5%)	20% Once daily, at night (4 weeks) perilesional petroleum jelly	Erythema, 15.6%; itching, 9.4%	

KOH: potassium hydroxide; NR: not reported/not specified; ^ the frequency of adverse events was calculated using the patients who completed the study as the denominator in the majority of studies, with a few exceptions (see text for further details); § 24 out of 26 patients included in the final analysis (percentage reported as 90% by the authors).

**Table 5:** Differences in the safety profile between KOH solution and active comparators: synopsis of results in the principal comparative studies.

First Author (Year)	Warts	KOH	Active Comparator	Difference Between Treatments in Side Effect Profile
Jayaprasad (2016) [22]	Plane (facial in 66.7%)	10% once weekly	TCA 30% once weekly	Subjective symptoms and hyperpigmentation more frequent with TCA (*p*-value NR)
Yaghoobi (2019) [25]	Plane (on head and neck in about two-thirds of patients)	10% once weekly	TCA 50% once weekly	Side effects more common with TCA, especially crust and dyspigmentation (*p*-value NR)
Alawady (2020) [27]	Plane (facial in 77%)	30% once weekly	TCA 30% once weekly	No significant differences, except for hypopigmentation, more common with KOH (*p* = 0.039)
Alawady (2022) [28]	Plane (predominantly facial)	30% once weekly	TCA 30% once weekly	No statistically significant differences (although rates were higher in the KOH group)
Hailu (2021) [37]	Anogenital	10% once daily	TCA 80% once weekly	Pain less severe in the KOH group (*p* < 0.05)
Farouk (2025) [31]	Plantar	40% once weekly	TCA 40% once weekly	No statistically significant difference in the incidence or type of adverse events
Galal (2020) [26]	Plane	5% once daily	Cryotherapy	No statistically significant differences, except for crust and scar, with a higher rate for cryotherapy (*p* < 0.05)
Al-Akkam (2018) [24]	Plane (facial)	5% once daily	Tretinoin 0.05% cream once daily	Side effects more frequent with KOH (*p*-value NR)
Yaghoobi (2014) [33]	Genital in males	5% twice a day	Cryotherapy	Significantly more adverse effects with KOH (*p* = 0.023 for burning, *p* = 0.0001 for ulceration)
Camargo (2014) [34]	Genital in males	5% daily	Cryotherapy	Superficial erosions more common with KOH; pain and hypopigmentation more frequent with cryotherapy (*p*-value NR)
Wickremasinghe (2010) [39]	Common (facial and genital excluded)	5% or 10% once daily	Cryotherapy	Hypopigmentation—especially pain—more frequent with cryotherapy; erythema more frequent with KOH (especially 10%) (*p*-value NR)
Javame Ghazvini (2025) [38]	Genital	5% once daily	Cryotherapy	No statistically significant difference
Işik (2014) [35]	Anogenital	5% once daily	FU+SA once daily	No significant difference (*p*-value NR)
Asadi (2016) [36]	Genital in females	5% twice a day	CO_2_ laser	No statistically significant difference
Hassan (2024) [43]	Common (not specified)	15% once daily	Hydrogen peroxide 20% once daily	No significant difference (*p*-value NR)

FU+SA: 0.5% 5-fluorouracil and 10% salicylic acid combination; KOH: potassium hydroxide; NR: not reported; TCA: trichloroacetic acid.

## Data Availability

Data will be made available by the authors upon request.

## Supplementary Materials

The following supporting information can be downloaded at: https://www.mdpi.com/article/10.3390/jcm15218592/s1, Supplementary Table S1. Full search strings for each consulted database.Supplementary Table S2. Risk of bias assessment and quality of included studies.

## Abbreviations

The following abbreviations are used in this manuscript:

KOH	Potassium hydroxide
HPV	Human papillomavirus
HIV	Human immunodeficiency virus
TCA	Trichloroacetic acid
FU+SA	Fluorouracil + salicylic acid
CO_2_	Carbon dioxide
