# Supplementary material for: Potassium Hydroxide Solution for the Treatment of Cutaneous and Anogenital Warts: A Narrative Review
Source: J Clin Med. 2026 Nov 4;15(21):8592. doi: 10.3390/jcm15218592 (PMC13763634; doi:10.3390/jcm15218592)
Supplement: Supplementary file 1 [file jcm-15-08592-s001.zip › jcm-4625121-supplementary.pdf]

Supplementary Table S1. Full search strings for each consulted database.

Database	Search strategy
**PubMed**	**1.** "Warts"[MeSH Terms]<br>**2.** wart[tiab] OR warts[tiab]<br>**3.** "condyloma acuminata"[tiab] OR "condylomata acuminata"[tiab]<br>**4.** 1 OR 2 OR 3<br>**5.** "Potassium Hydroxide"[MeSH Terms]<br>**6.** KOH[tiab] OR "potassium hydroxide"[tiab]<br>**7.** 5 OR 6<br>**8.** 4 AND 7
**Ovid MEDLINE**	**1.** exp Warts/<br>**2.** exp Condylomata Acuminata/<br>**3.** (wart* or condyloma acuminata or condylomata acuminata).ti,ab,kf.<br>**4.** or/1-3<br>**5.** Potassium Hydroxide/<br>**6.** exp Hydroxides/<br>**7.** Potassium Compounds/<br>**8.** (KOH or potassium hydroxide).ti,ab,kf.<br>**9.** or/5-8<br>**10.** 4 and 9
**Google Scholar**	"potassium hydroxide" OR KOH warts OR wart OR condylomata
**Embase.com**	**1.** 'wart'/exp<br>**2.** 'condyloma acuminatum'/exp<br>**3.** wart*:ti,ab,kw OR 'condyloma acuminata':ti,ab,kw OR 'condylomata acuminata':ti,ab,kw<br>**4.** 1 OR 2 OR 3<br>**5.** 'potassium hydroxide'/exp<br>**6.** koh:ti,ab,kw OR 'potassium hydroxide':ti,ab,kw<br>**7.** 5 OR 6<br>**8.** 4 AND 7
**Scopus**	(("Warts" OR "Wart" OR "Condylomata acuminata" OR "Condyloma acuminata") AND ("potassium hydroxide" OR "KOH"))

Supplementary Table S2. Risk of bias assessment and quality of included studies.

				**Non-randomized studies[a]**				
			**Selection**		**Comparability**		**Outcome**	**Overall quality**
Al-Hamdi 2012			**		***		*	**M**
Rezk 2017			*		**		*	**L**
Al-Akkam 2018			**		**		*	**M**
Galal 2020			*		**		*	**L**
Alawady 2020			*		**		*	**L**
Loureiro 2008			*		*		*	**L**
Asadi 2016			**		**		*	**M**
Hailou 2021			*		**		*	**L**
Kandil 2016			**		**		*	**M**
Makki 2022			*		*		*	**L**
Hassan 2024			*		**		*	**L**
				**Randomized controlled trials[b]**				
	**1**	**2**	**3**	**4**	**5**	**6**	**7**	**Overall risk of bias**
Jayaprasad 2016	**U**	**U**	**H**	**H**	**M**	**L**	**M**	**M**
Yaghoobi 2019	**U**	**U**	**M**	**M**	**L**	**U**	**M**	**M**
Alawady 2022	**U**	**U**	**U**	**U**	**U**	**U**	**H**	**H**
Hanif 2022	**U**	**U**	**H**	**H**	**L**	**U**	**M**	**M**
Bashir 2024	**U**	**U**	**H**	**H**	**L**	**U**	**M**	**M**
Farouk 2025	**U**	**U**	**H**	**L**	**L**	**L**	**M**	**L**
Yaghoobi 2014	**U**	**U**	**H**	**H**	**H**	**U**	**H**	**H**
Camargo 2014	**H**	**H**	**H**	**H**	**H**	**U**	**M**	**H**
Isik 2014	**M**	**U**	**H**	**H**	**U**	**U**	**M**	**M**
Javame Ghazvini 2025	**M**	**U**	**H**	**H**	**L**	**L**	**M**	**M**
Wickremasinghe 2010	**U**	**U**	**H**	**H**	**U**	**U**	**M**	**H**
Elgayar 2025	**U**	**U**	**H**	**L**	**L**	**L**	**M**	**L**
Kalfa 2024	**U**	**U**	**H**	**H**	**U**	**U**	**M**	**H**

L, low; H, high; U, unclear; M, moderate.

[a] Study quality assessment performed by means of Newcastle/Ottawa scale (each asterisk represents if the respective criterion within the subsection was satisfied)

[b] Cochrane Collaboration's tool (Rob1) for assessing the risk of bias across 7 domains: 1 (Random sequence generation), 2 (Allocation concealment), 3 (Blinding of participants and personnel), 4 (Blinding of outcome assessment), 5 (Incomplete outcome data), 6 (Selective reporting) and 7 (Other bias).
